# Vacuum Interfacial Structure and X-ray Reflectivity of Imidazolium-Based Ionic Liquids with Perfluorinated Anions from a Theory and Simulations Perspective

**DOI:** 10.1021/acs.jpcc.2c03311

**Published:** 2022-08-05

**Authors:** Waruni V. Karunaratne, Man Zhao, Edward W. Castner, Claudio J. Margulis

**Affiliations:** † Department of Chemistry, University of Iowa, Iowa City, Iowa 52242, United States; ‡ Department of Chemistry and Chemical Biology, Rutgers, The State University of New Jersey, Piscataway, New Jersey 08854, United States

## Abstract

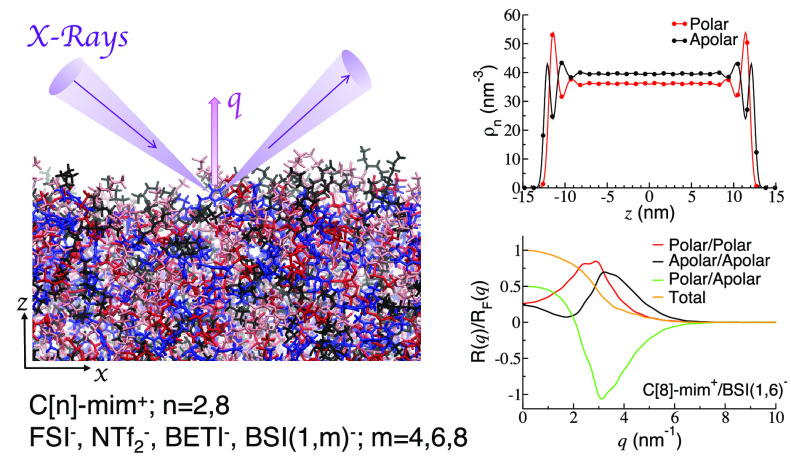

We report studies of the vacuum interfacial structure of a series of 1-methyl-3-alkylimidazolium bis(perfluoroalkanesulfonyl)imide ionic liquids (ILs) and predict and explain their Fresnel-normalized X-ray reflectivity. To better interpret the results, we use a theory we recently developed dubbed “the peaks and antipeaks analysis of reflectivity” which splits the overall signal into that of different pair subcomponents. Whereas the overall reflectivity signal is not very informative, the peak and trough intensities for the pair subcomponents provide rich information for analysis. When species containing cationic alkyl or anionic fluoroalkyl tails are present at the interface, a tail layer is found next to a vacuum, and this tail layer can be composed of both alkyl and fluoroalkyl moieties. To maintain the positive–negative alternation of charged groups, alkyl and fluoroalkyl tails must necessarily be nearby and cannot segregate. Charged groups are found in the subsequent layer just below the interface and arranged to achieve lateral charge neutrality. In general, fluctuations at and away from the interface are based on polarity (i.e., heads and tails) and not on charge; when there are no significant alkyl or fluoroalkyl moieties in the IL, atomic density fluctuations away from the interface are small and appear to exist for the purpose of achieving lateral charge balance. For all the systems reported here, the persistence length of density fluctuations does not go beyond ∼7 nm.

## 1 Introduction

In many of the practical areas where ionic liquids (ILs) find application, they are exposed to interfaces. For prototypical ionic liquids with polar heads and apolar alkyl tails the picture at the vacuum interface is simple—apolar tails point outward at the interface. However, the situation when different size alkyl and fluoroalkyl tails are considered (including when there are no apolar moieties) is less clear. Of the several techniques that can probe the IL/vacuum interface,^1−7^ our computational study focuses on specular X-ray reflectivity (XRR). Because of the significant extent of X-ray penetration, this technique can provide details about a system from the interface to deep into the bulk liquid phase.^8^

For a multicomponent system the interpretation of the often feature-poor Fresnel-normalized reflectivity can be difficult. One can think of an IL as having two components based on the ions or four if subdividing the ions into positive, negative, apolar alkyl, and apolar fluoroalkyl moieties. Even when reasonable inversion of the reflectivity to obtain an average electron density at and away from the interface can be accomplished, assigning contributions to each of the multiple different subspecies is challenging. We present in this work a practical implementation of theoretical methodology we recently developed^9,10^ to better understand X-ray reflectivity from molecular dynamics (MD) simulations in the hope to encourage more colleagues working on ILs to attempt the technique as part of their structural studies.

The persistence length for density oscillations due to broken symmetry for ILs at an interface has been a topic of interest for a long time now, especially considering some puzzling experimental findings.^11−16^ ILs under confinement have already been studied by using the reflectivity technique; for example, Haddad et al.^17^ studied the air/IL interfacial structure for 1-alkyl-3-methylimidazolium bis(trifluoromethylsulfonyl)imide (C[*n*]-mim^+^/NTf_2_^–^) as a function of alkyl tail length (*n* = 2–22). Similarly at a vapor interface, Mezger and co-workers^18^ studied the temperature-dependent structure of C[22]-mim^+^/NTf_2_^–^ using X-ray reflectivity and grazing incidence scattering experiments. The interfacial structure of C[2]-mim^+^/NTf_2_^–^ at a sapphire surface was also investigated by using X-ray reflectivity in conjunction with MD simulations.^19^ Our group has also investigated ILs under confinement^9,20−22^ using MD simulations, and in ref (9) we developed the peaks and antipeaks analysis for reflectivity; the ideas introduced in ref (9) were later expanded and applied to the study of diamond-confined molten alkali chloride salts.^10^ The literature on the structure of ILs using other surface sensitive techniques is vast, and a full set of citations is beyond the scope of this work, but particularly important has been the pioneering work by Baldelli et al. using sum frequency generation^23−27^ as well as Perkin et al.^28,29^ and Atkin et al. using force microscopy.^30−33^

**Figure 1:**
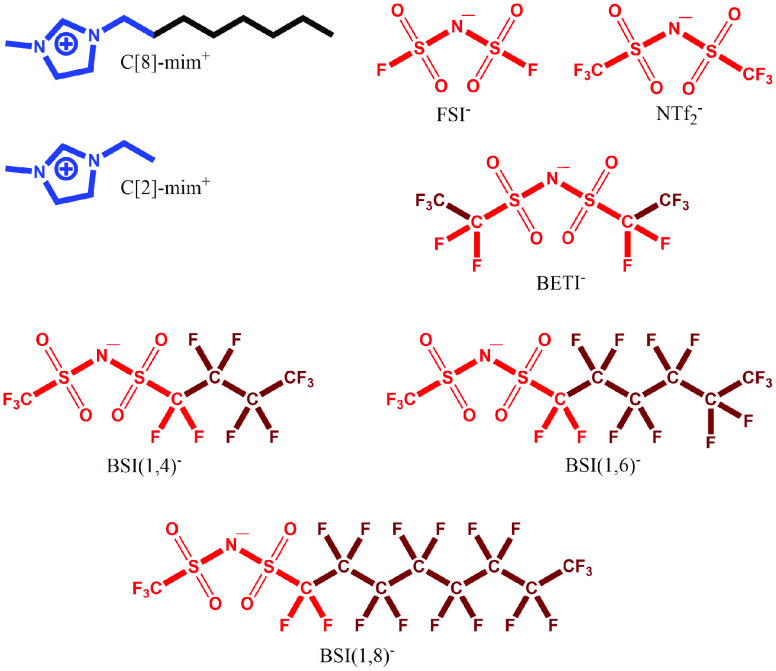
Chemical structures of ions used in this study. For each IL, the C[8]-mim^+^ or C[2]-mim^+^ cation is combined with one of the depicted anions. The moieties that we label positive, negative, alkyl (Apolar_C/H_) and fluoroalkyl (Apolar_C/F_) in the text are denoted in this figure in blue, red, black, and brown, respectively.

The present study focuses on the interfacial behavior of a series of ILs based on the C[8]-mim^+^ and C[2]-mim^+^ cations coupled with the bis(perfluoroalkanesulfonyl)imide (BSI) family of anions. Specifically our simulations are for the two cations combined with bis(fluorosulfonyl)imide (FSI^–^), bis(trifluoromethylsulfonyl)imide (NTf_2_^–^), bis(perfluoroethylsulfonyl)imide (BETI^–^), (trifluoromethylsulfonyl) (perfluorobutylsulfonyl)imide (BSI(1,4)^−^), (trifluoromethylsulfonyl)(perfluorohexylsulfonyl)imide (BSI(1,6)^−^) and (trifluoromethylsulfonyl)(perfluorooctylsulfonyl)imide (BSI(1,8)^−^) for which some of the physical properties in the bulk liquid phase were reported in a previous publication.^34^ To the best of our knowledge, ILs with the BSI(1,6)^−^ and BSI(1,8)^−^ anions have not yet been synthesized but are included here to obtain a better perspective on what happens when the fluoroalkyl tail becomes longer. Whereas ILs with fluorous anions like NTf_2_^–^ have been widely studied because they make good battery materials and have low viscosities, the interfacial behavior of ILs with other fluoroakyl tails is less understood, and even less is known about their interfacial behavior when in combination with cations of long and short alkyl tails. As it will become clear in the next sections, the extent to which alkyl and fluoroalkyl tails are exposed to the interface will be determined by the driving force of charged cationic and anionic heads to create an electroneutral layer that is perpendicular to the surface normal; because charged species seek to stay in “a layer”, alkyl and fluoroalkyl tails of different lengths will have to be exposed to a vacuum to different extents. Chemical structures of all ions used in this study are shown in Figure 1.

## 2 Computational Methods

All molecular dynamics simulations were performed with the GROMACS^35−37^ code using the Canongia-Lopes and Pádua (CL&P)^38−41^ and optimized potentials for liquid simulations all-atoms (OPLS-AA)^42−45^ force fields. All atomic partial charges were scaled to 78% of the nominal charge to better match the experimentally reported structure and transport properties of C[8]-mim^+^/NTf_2_^–^ and C[2]-mim^+^/NTf_2_^–^. For these systems, a comparison between experimental and simulated structure functions (*S*(*q*)), densities, and viscosities is provided in Section S.2.

Simulations in this study were performed by using the leapfrog algorithm^46^ with a time step of 1 fs to integrate the equations of motion, except during the initial energy minimization step where the steepest descent algorithm was used. To compute the electrostatic interactions, the particle-mesh Ewald (PME) method^47,48^ with a Fourier grid spacing of 0.08 nm and a B-spline interpolation of order 6 was used (see below for further details about the slab configuration). Short-range Coulomb and Lennard-Jones cutoffs were maintained at 1.5 nm in all simulations; long-range energy and pressure corrections were done for bulk simulations but not for slab simulations. At this point, it is worth mentioning that there is a correction available for interactions between alkyl and fluoroalkyl systems in the literature.^49^ In the absence of strong Coulombic interactions, the pattern of organization of alkyl and fluoroalkyl systems may be altered by such types of corrections; however, we tested simulations for ILs with these parameters both in the bulk and at interfaces (data not shown) and found that changes in our results were negligible. Therefore, results presented here are those that do not include these corrections. The key point here is that because ILs are dominated by positive–negative charge interactions, on a molecular level alkyl and fluoroalkyl tails will have to organize decorating the charge network in an alternating pattern as well.

### Bulk ILs Simulations

First, for each IL a rectangular shape simulation box containing 2000 ion pairs (Figure 1) was generated by using the FFTOOL^50^ and PACKMOL^51^ packages. Following steps similar to those in our previous study,^9^ these initial configurations were energy minimized and then equilibrated at 300 K in the isobaric–isothermal (NPT) ensemble for 4.2 ns while gradually increasing charges from 1% to 100% (of the intended 78% target value) and decreasing the pressure from 50 to 1 bar by using the V-rescale^52^ thermostat and the Berendsen^53^ barostat as coded in GROMACS. In subsequent steps, when a thermostat was required, we used that by Nosé and Hoover,^54−56^ and when a barostat was used, it was that introduced by Parrinello and Rahman^57^ with time constants 0.2 ps for the thermostat and 1.0 ps for the barostat. For each system, during an 8 ns simulated annealing run in the NPT ensemble, the temperature was ramped from 300 to 600 K and back to 425 K; this step was then followed by a 100 ns bulk production run at that final temperature and in the same ensemble. The last 20 ns of this production run was later used to compute *S*(*q*) at 425 K; these functions are displayed in Figure S4. Before beginning our interfacial simulations, systems were further equilibrated in the constant volume and constant temperature (NVT) ensemble for another 5 ns at the same temperature. During bulk phase simulations, the EW3D PME method was used as coded in GROMACS.

### Interface Simulations

**Figure 2:**
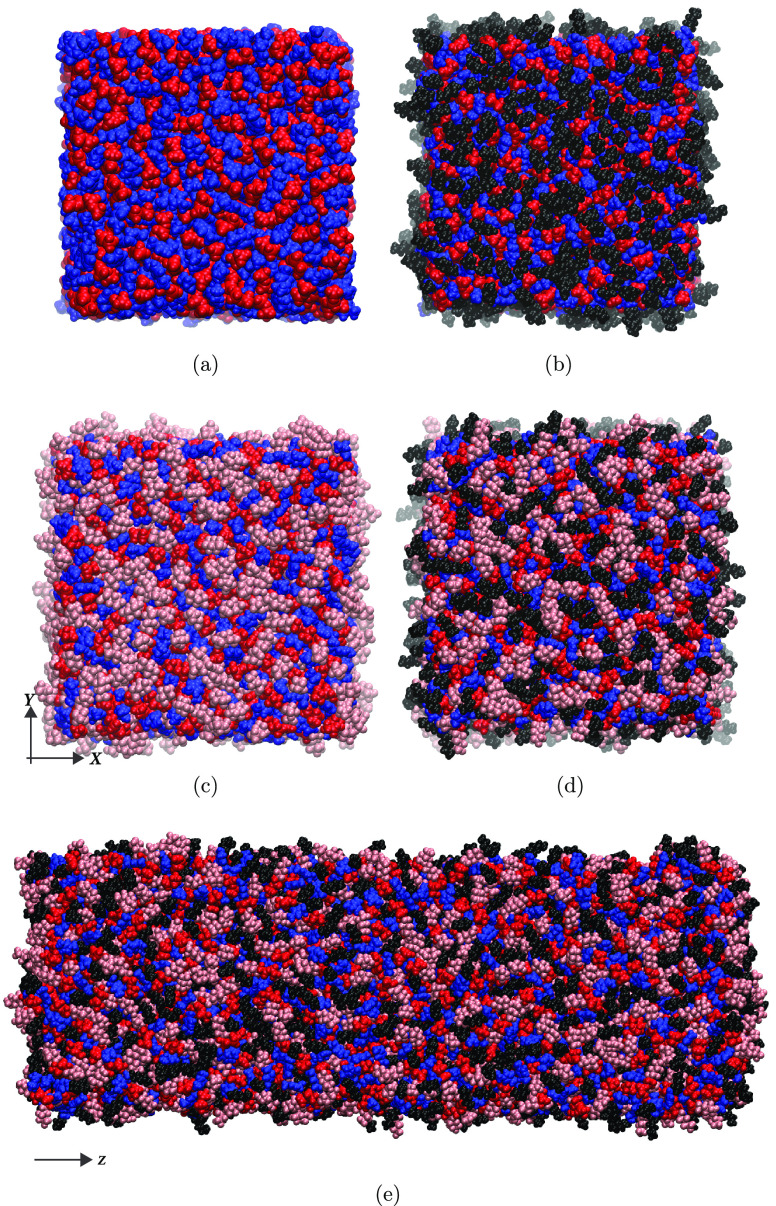
Top view of final simulation snapshots (i.e., *z*-axis of the simulation box is perpendicular to the page) for vacuum-confined (a) C[2]-mim^+^/FSI^–^, (b) C[8]-mim^+^/FSI^–^, (c) C[2]-mim^+^/BSI(1,6)^−^, and (d) C[8]-mim^+^/BSI(1,6)^−^; (e) shows a side view of the C[8]-mim^+^/BSI(1,6)^−^ vacuum-confined system. Here the *z*-axis of the simulation box lies parallel to the page. In all plots, positive = blue, negative = red, alkyl = black, and fluoroalkyl = rose. Side views of systems (a) through (c) are provided in Figure S1.

For all interfacial simulations (which were run in the NVT ensemble), we used the Yeh–Berkowitz correction^58,59^ (EW3DC) to approximate the 2D Ewald summation in the slab geometry. For this purpose, the final snapshot from each bulk simulation was introduced at the center of a rectangular supercell with sufficient vacuum (see Table S3) to properly apply the EW3DC method; these vacuum slabs confined the IL from both ends on the *z* direction. The vacuum-confined systems were relaxed for 5 ns at 425 K followed by an 8 ns simulated annealing run that gradually ramped up the temperature to 600 K and brought it back to a value of 425 K. For each system, the final production run in the slab configuration performed at the same temperature was 200 ns in duration, and the last 180 ns of those trajectories was used for analysis. Figures 2 and S1 show final simulation snapshots for a few selected vacuum/IL systems. Further size details for each simulation are provided in Table S3.

## 3 Results and Discussion

This section starts with a discussion of ionic and subionic (positive, negative, alkyl, fluoroalkyl, polar, and apolar) number density profiles along the surface normal *z* direction and follows with a description of the orientation of the ions as a function of the distance to the interface. The section concludes with our peaks and antipeaks analysis of the predicted Fresnel-normalized X-ray reflectivity. We highlight here some important points for others seeking to do similar studies on IL systems. First, we want to explain why our interfacial simulations are so long; 200 ns at 425 K may seem unnecessary to get at bulk properties but this is not the case for interfacial properties.^9,20^ Ionic liquids are naturally nanopatterned materials with polar networks and apolar domains. While these patterns have no specific orientation in the bulk liquid phase, the computation of interfacial number density profiles over a short time interval will necessarily show peaks and troughs corresponding to this pattern of nanostructures from the vacuum to the bulk phase. Such a transient pattern of peaks and troughs is not useful for our study since we only care about the more “permanent” density fluctuations with specific orientation, defined by the interface, due to broken symmetry. Our interfacial simulations must be long enough so that we average over the lifetime of the bulk nanostructures leaving only those persistent structural oscillations that occur because of the interface. In other words, our density profiles should be flat if there is no interface-created liquid structure or when deep in the bulk phase.

Second, we comment here on our choice^10^ for analysis based on number density profiles and not electron density profiles or the actual charge density profiles of the liquid. In the XRR literature it is often the electron density profile that is plotted because the technique is sensitive to that; two systems can have identical or nearly identical atomic density profiles but very different X-ray reflectivities. Of course, the peaks and antipeaks analysis of reflectivity takes this into account, but we prefer to keep our discussion focused as much as possible on the ionic and subionic number density profiles because we believe that scientists interested in interfacial structure care most about the organization of the ions as opposed to the overall electron density of the liquid.

### Real Space Analysis of the Interfacial Structure

**Figure 3:**
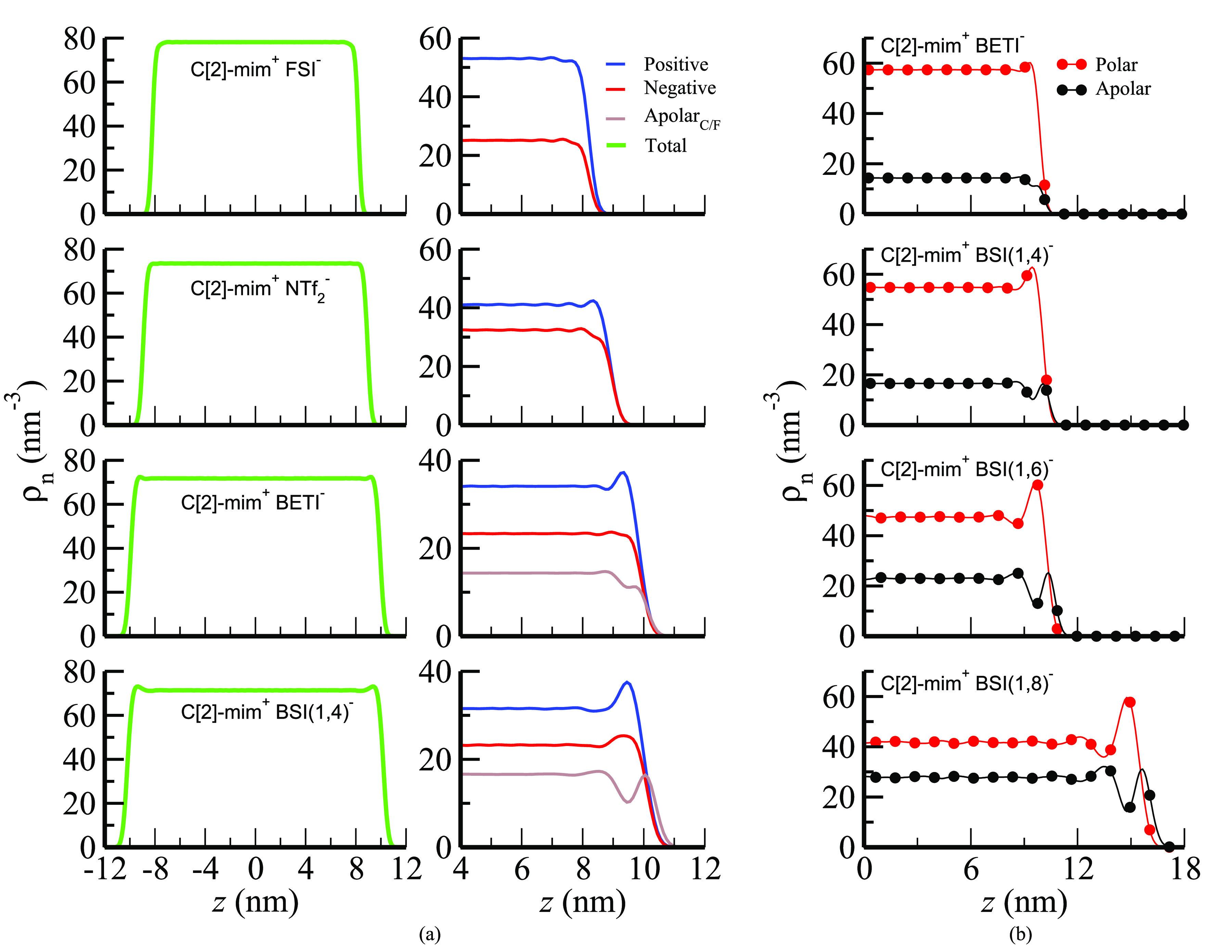
Symmetrized number density profiles for vacuum-confined C[2]-mim^+^-based ILs; “0” nm coincides with the center of the simulation box: (a) full system (left); positive, negative, and apolar fluorocarbon subcomponents (right) (corresponding plots for C[2]-mim^+^/BSI(1,6)^−^ and C[2]-mim^+^/BSI(1,8)^−^ are provided in Figure S5a). (b) Density profiles of polar (positive and negative combined) and apolar (fluorocarbon tails) for ILs with the BETI^–^ through BSI(1,8)^−^ anions.

For systems like C[2]-mim^+^/FSI^–^ and C[2]-mim^+^/NTf_2_^–^ in which ions are smaller and with no apolar parts, properly equilibrated liquid density profiles are essentially flat everywhere except at the immediate interface where these monotonically grow from or decrease to zero; this can be clearly seen from Figure 3a (left). Similar behavior has been observed experimentally for C[2]-mim^+^/NTf_2_^–^ at 298 K in ref (17). If for systems with these smaller ions and no apolar tails one splits the overall density profile in terms of that of the positive and negative atomic subcomponents (as in Figure 1), a pattern of oscillations emerges as can be gleaned from Figure 3a (right). These small oscillations due to ion size and shape mismatch are to some extent offset when comparing cations and anions. Assuming from such finding that this is due to preference for cationic or anionic charge at the vacuum interface would be incorrect since the actual positive and negative charge densities (as opposed to the atom number density profiles of the positive and negative subcomponents) are for the most part aligned (data not shown). In other words, it appears that for these smaller ions the cationic and anionic density fluctuations may be there not because there is a preference for cations or anions at and away from the interface but instead as an attempt to preserve lateral charge neutrality.

For the C[2]-mim^+^ cation coupled with the BSI(1,4)^−^, BSI(1,6)^−^, and BSI(1,8)^−^ anions, the immediate vicinity of the vacuum interface is dominated by the fluoroalkyl tail layer followed on the liquid side by a layer of polar components. When positive and negative heads are considered together as the polar component, what we find is the expected polar–apolar segregation at the interface as can be gleaned from Figure 3b. Notice also how for C[2]-mim^+^/BSI(1,4)^−^ in Figure 3a (right) as well as for C[2]-mim^+^ coupled with BSI(1,6)^−^ and BSI(1,8)^−^ in Figure S5a the positive and negative atomic number density peaks appear at about the same distance from the interface. This highlights that these systems also nearly maintain in-plane charge neutrality. We will see that this is common across all systems studied here, and it will affect most prominently the exposure of alkyl and fluoroalkyl tails at the interface when these are of distinct lengths.

**Figure 4:**
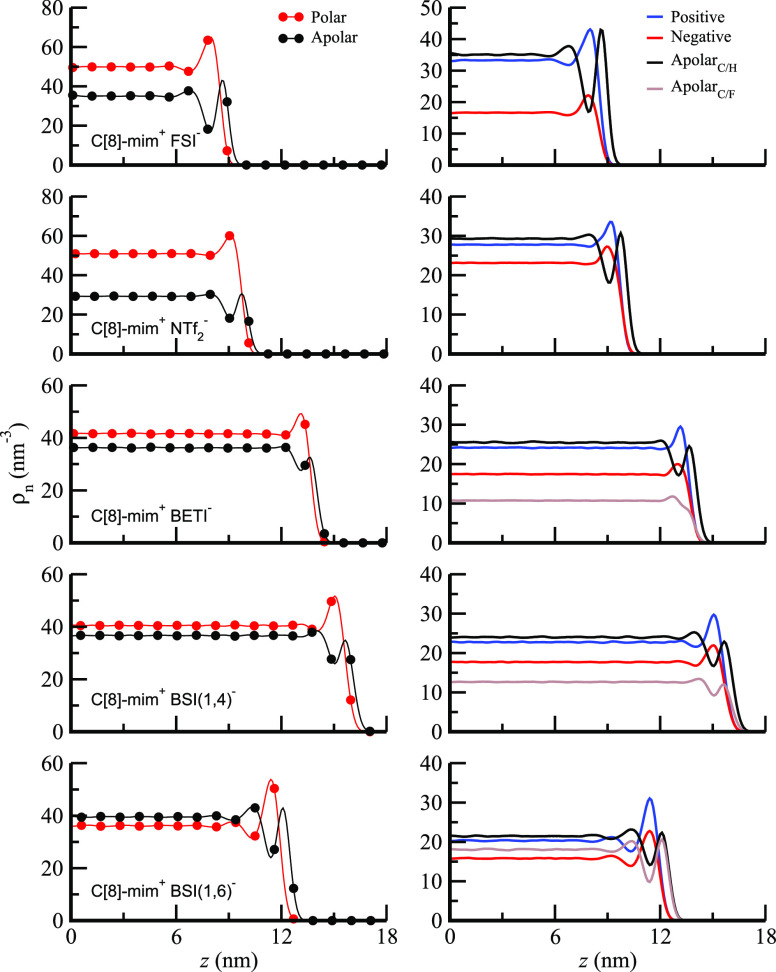
Symmetrized number density profiles for vacuum-confined C[8]-mim^+^/FSI^–^ through C[8]-mim^+^/BSI(1,6)^−^ (see Figure S6 (left) for C[8]-mim^+^/BSI(1,8)^−^) with “0” nm coinciding with the center of the simulation box. (left) Polar and apolar subcomponents; (right) positive, negative, apolar hydrocarbon (Apolar_C/H_), and apolar fluorocarbon (Apolar_C/F_) subcomponents.

Figures 4 and S6 (left) show number density profiles of polar and apolar as well as positive, negative, alkyl, and fluoroalkyl subcomponents for salts containing the C[8]-mim^+^ cation. In this case, apolar refers to the combination of alkyl and fluoroalkyl moieties as defined in Figure 1. When the counterion of C[8]-mim^+^ is small, the pattern is consistent with that in Figure 3 for C[2]-mim^+^ coupled with the longer fluoroalkyl tail anions in that the alkyl tail of C[8]-mim^+^ tends to protrude at the interface and a polar layer occurs deeper in the liquid where positive and negative moieties are at about the same *z* distance from the interface.

Things become more interesting when we consider C[8]-mim^+^ coupled with the larger fluoroalkyl anions; here again positive and negative components have peaks at around the same *z* value, implying that it is lateral charge neutrality and the necessity to keep charges away from the vacuum which drives the liquid arrangement at the interface. Notice that because of lateral charge alternation, alkyl tails and fluoroalkyl tails must necessarily coexist in contact at the ionic level. In other words, there cannot be alkyl and fluoroalkyl segregation at the interface if positive and negative charges must be adjacent. The extent to which alkyl and fluoroalkyl tails protrude at the interface depends on their length. Because it is lateral charge ordering and not apolar aggregation that truly defines the *z* depth of the ions, those with longer tails will protrude more significantly toward the vacuum. In other words, cations or anions will not align terminal tail groups at the interface, but instead charge head groups deeper in the liquid phase will be aligned in plane (or in a slab to be most precise). This can be clearly seen from the fact that for C[8]-mim^+^/BSI(1,4)^−^ it is the alkyl groups that dominate the immediate vicinity with the vacuum whereas for C[8]-mim^+^/BSI(1,8)^−^ it is the fluoroalkyl groups.

**Figure 5:**
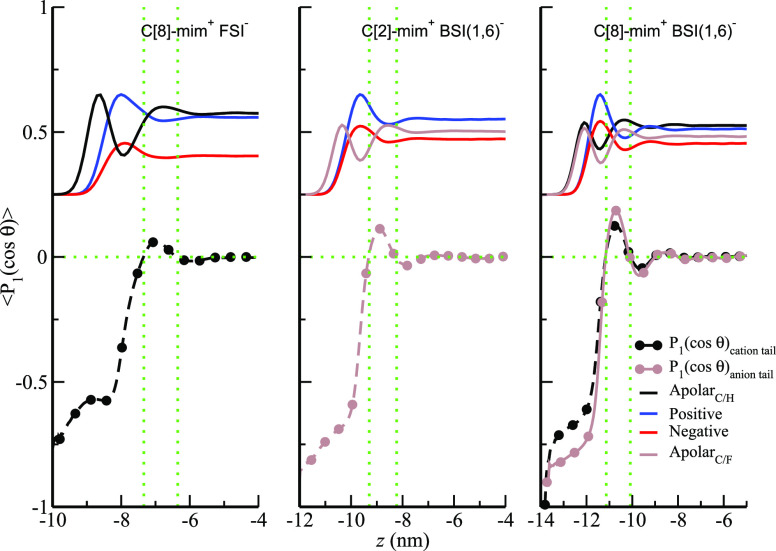
Orientational order parameter, *P*_1_(cos(θ)), as a function of *z* for C[8]-mim^+^/FSI^–^, C[2]-mim^+^/BSI(1,6)^−^, and C[8]-mim^+^/BSI(1,6)^−^ along with their corresponding number density profiles (vertically shifted and not to scale with the *y*-axis). Negative values of *P*_1_(cos(θ)) correspond to outward pointing tails; *z*-axis direction defined as in Figure 2, and *z* = 0 corresponds to the center of the simulation box.

So far we have discussed the immediate space in contact with a vacuum, but if we look carefully particularly in the case of the larger ions, second and even third peaks away from the interface can be observed in density profiles corresponding to layers of polar and apolar subcomponents. Such layers are better understood by analyzing the *z*-dependent angular orientation of the ions in combination with the atomic density profiles. For this we defined a unit vector pointing from the fourth-from-terminal to the terminal tail carbon atom and an angle θ between this unit vector and the *z*-axis as defined in Figure 2. With these definitions we computed the average of the first Legendre polynomial *P*_1_(cos(θ)); a negative value for *P*_1_(cos(θ)) implies that tails point outward in the direction of the interface, and the opposite is true for a positive value. Figure 5 shows plots of *P*_1_(cos(θ)) computed for cation and anion tails in C[8]-mim^+^/FSI^–^, C[2]-mim^+^/BSI(1,6)^−^, and C[8]-mim^+^/BSI(1,6)^−^. Overlayed on top of these figures are corresponding number density profiles. In all of these graphs we find the outward tail orientation at the interface and a shift to the inward pointing orientation deeper in the liquid phase. Depending on the system, we may then find another outward pointing layer beyond which *P*_1_(cos(θ)) = 0, which is an indication that we have reached the bulk liquid phase. A key point in these figures is that the cationic and anionic heads associated with the first peak in the corresponding number density profiles must necessarily account for both ions in the apolar layer forming at the interface and those forming an incipient bilayer deeper in the liquid. For all of these systems, even for the larger ions, the structural features at the interface are limited to about ∼4–7 nm depending on the combination of the IL anions and cations; beyond this distance density profiles and angular distributions become for the most part flat indicating that bulk characteristics have been achieved.

### Reciprocal Space Analysis of the Interfacial Structure Using X-ray Reflectivity

**Figure 6:**
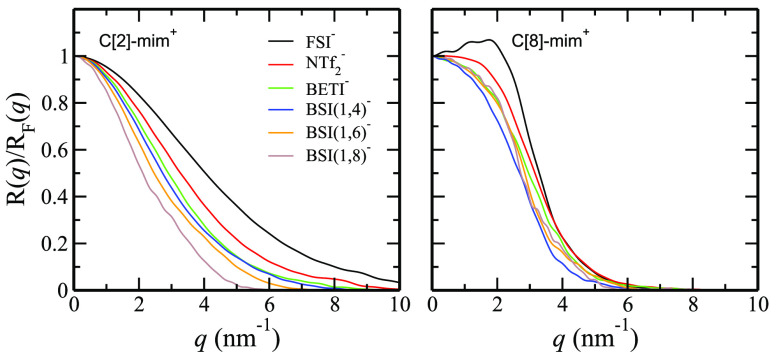
Total Fresnel-normalized specular X-ray reflectivity, , versus scattering vector along the surface normal direction, *q*. C[2]-mim^+^-based ILs show a clear trend for different anions—the function decays at lower *q* values as the anion becomes larger—but this is not the case in the case of the C[8]-mim^+^-based ILs.

Figure 6 shows the Fresnel-normalized reflectivity  across systems. One cannot help but notice that these curves are mostly featureless and that all information about the differences across salts is concealed in the width and shape of otherwise qualitatively similar functions. Across the family of C[2]-mim^+^ containing ILs,  decays to low intensity at smaller *q* values when the fluoroalkyl tails of the anions are longer, but no such trend is observed for the C[8]-mim^+^ containing ILs. We ascribe this behavior to the fact that for C[2]-mim^+^ ILs the liquid interfacial region (specifically the apolar component) grows with the size of the anionic tail, but for C[8]-mim^+^ ILs this is not necessarily the case and multiple combined effects influence the reflectivity (*vide infra*).

**Figure 7:**
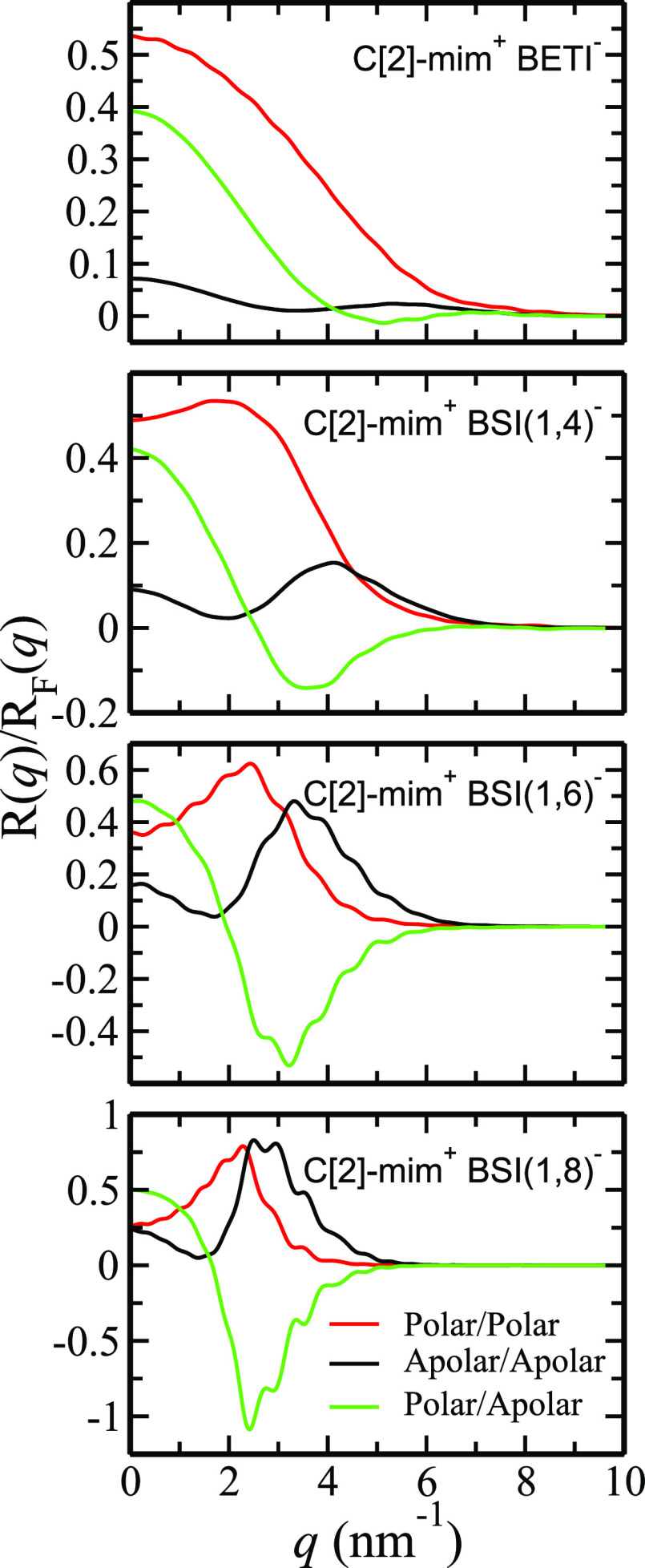
Reflectivity, , partial subcomponents for the family of C[2]-mim^+^-based ILs combined with anions defined as having a fluoroalkyl tail in Figure 1.

The peaks and antipeaks analysis of reflectivity helps dissect the almost featureless  functions into subcomponents that actually have peaks and troughs at specific *q* values of relevance. The locations of these peaks and troughs help us better understand what different species are structurally doing at and away from the interface. Our analysis is based on species pairs irrespective of their location (at the interface or deep in the liquid phase) and is different in philosophy from other commonly used partitions of the normalized reflectivity based on layers. In this work we will use two different splitting schemes for the species giving rise to ; the first one is based on correlations between polar and apolar subcomponents as in eq 1 (here polar refers to the combined contribution of cation and anion heads whereas apolar refers to the combined contribution of cation and anion tails all as defined in Figure 1), and the second is based on correlations between each of the different subionic subcomponents (positive, negative, alkyl, and fluoroalkyl) as in eq 2. The reader is referred to refs (9) and (10) for the analytical derivation of equations associated with the peaks and antipeaks analysis of reflectivity that we seek not to repeat here.
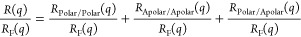
1
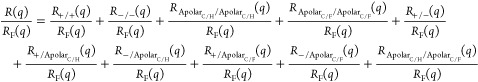
2The case of the C[2]-mim^+^-based ILs is the simplest to understand; Figure 7 shows the polar–polar, polar–apolar, and apolar–apolar components of  for C[2]-mim^+^/BETI^–^, C[2]-mim^+^/BSI(1,4)^−^, C[2]-mim^+^/BSI(1,6)^−^, and C[2]-mim^+^/BSI(1,8)^−^. We first consider C[2]-mim^+^/BSI(1,8)^−^ for which two peaks and one antipeak are clearly observed in Figure 7. The peaks are for “same-type” correlations and the antipeak for “different-type” correlations; in other words, polar–polar correlations and apolar–apolar correlations appear as peaks whereas polar–apolar correlations as an antipeak. Notice that the red line corresponding to the polar correlations is shifted to lower *q* values when compared to the black line corresponding to the apolar correlations; this implies that the polar layer is deeper inside the liquid phase and that the main apolar layer is the one protruding toward the vacuum. The reason the green line associated with the polar–apolar structural correlations shows as an antipeak is that there is a spatial offset between polar and apolar moieties. The case of C[2]-mim^+^/BSI(1,6)^−^ is not too different from that of C[2]-mim^+^/BSI(1,8)^−^, but the pattern of two peaks and one antipeak starts changing as the fluoroalkyl tail becomes shorter and is mostly gone for C[2]-mim^+^/BETI^–^. This is because there is not much of an apolar layer or polar–apolar alternation at the interface for C[2]-mim^+^/BETI^–^. Notice how as we go from C[2]-mim^+^/BSI(1,8)^−^ to C[2]-mim^+^/BETI^–^ the subcomponents decay to zero at larger *q* values. Peaks at lower *q* value are indicative of correlations that happen at longer distances, and it is then no surprise that for C[2]-mim^+^/BSI(1,8)^−^ the polar peak is at a significantly lower *q* value than that for C[2]-mim^+^/BSI(1,4)^−^. It now makes sense that the overall  decays at lower *q* values for the larger anions coupled with C[2]-mim^+^ in Figure 6 because features move to lower *q* as the fluoroalkyl layer becomes more prominent.

**Figure 8:**
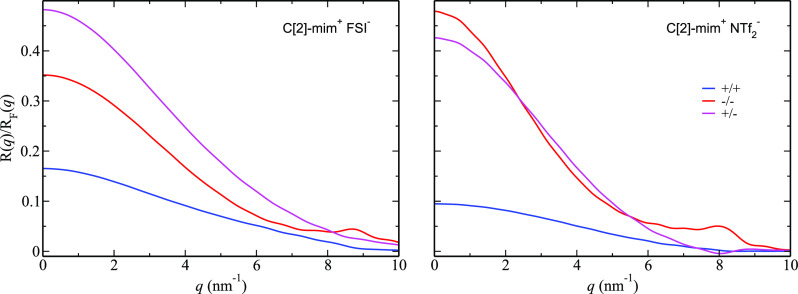
For C[2]-mim^+^/FSI^–^ and C[2]-mim^+^/NTf_2_^–^, partial subcomponents of  based on charge (see eq 2).

We focus now on C[2]-mim^+^/FSI^–^ and C[2]-mim^+^/NTf_2_^–^ which have no apolar components. If we split  in terms of the pair correlations of charged subcomponents as defined in Figure 1, the only interesting features we observe in Figure 8 are small peaks and antipeaks at or above 8 nm^–1^; notice that these correspond to subnanometer distances in real space that are not associated with polar–apolar alternation but instead with the small offset in the real space oscillations in Figure 3a (right) for C[2]-mim^+^/FSI^–^ and C[2]-mim^+^/NTf_2_^–^. X-ray reflectivity is sensitive to the electronic density of the system which is modulated by oscillations in number density; whereas the oscillations in number density between cations and anions depicted in Figure 3a (right) are real and cause the peaks and antipeaks observed in Figure 8, they appear to be there solely for the purpose of achieving lateral charge balance and not because there is a preference for cations or anions at the interface. Notice also how in Figure 8 partial subcomponents involving the anion  contribute the most to the overall reflectivity signal. This results from the larger X-ray form factors from species with more electrons such as O, F, and S atoms in the anions, which provide better X-ray contrast when compared to H, C, and N atoms in the cations.

**Figure 9:**
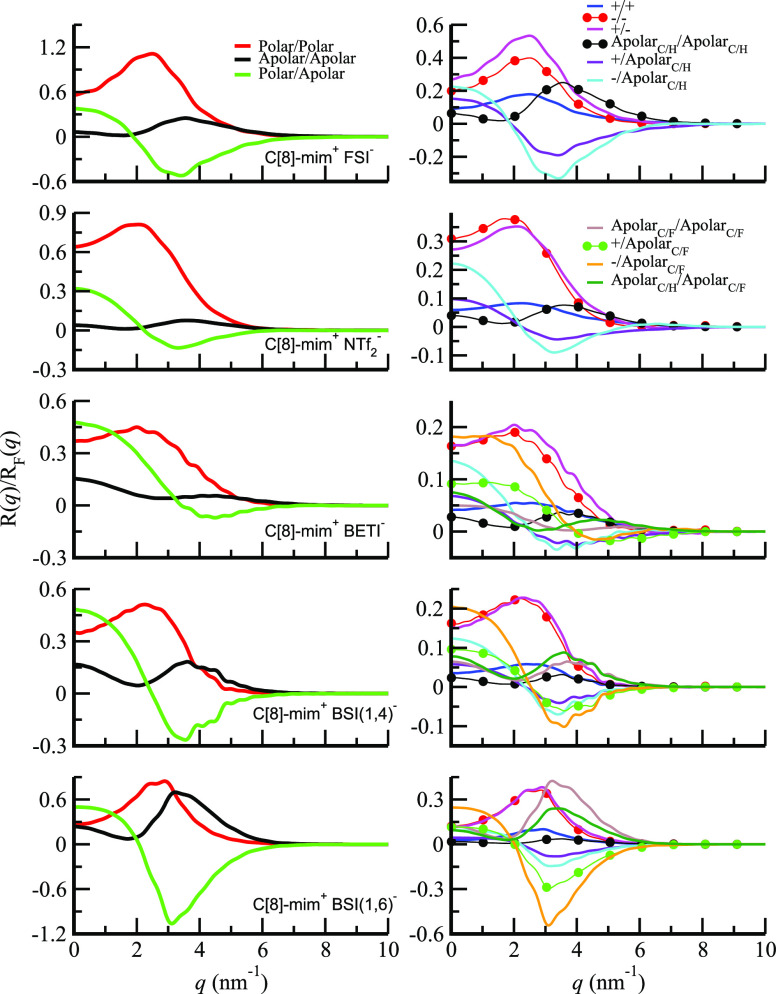
X-ray reflectivity partial subcomponents for C[8]-mim^+^-based ILs: (left) polarity partition of ; (right) positive, negative, alkyl, and fluoroalkyl based partition of . The case of C[8]-mim^+^/BSI(1,8)^−^ is presented in Figure S6 (right).

The trends in reflectivity for the family of C[8]-mim^+^ ILs are quite different from those we have thus far discussed because in all cases except when combined with BSI(1,8)^−^ (see Figure S6 (bottom left)) it is the octyl tail that defines the length of the apolar layer. This can be gleaned from Figure 4 (right) where the outermost moiety in contact with the vacuum is the alkyl tail of C[8]-mim^+^ when combined with FSI^–^, NTf_2_^–^, BETI^–^, and BSI(1,4)^−^, whereas for C[8]-mim^+^/BSI(1,6)^−^ both alkyl and fluoroalkyl tails have about the same length and protrude equally at the interface. For the C[8]-mim^+^-based ILs coupled with different fluorous anions, Figure 9 shows the partial subcomponents of  based on eq 1 on the left panel and eq 2 on the right panel (see also Figure S6 (right)). We start our discussion with the polarity partition on the left panel of Figure 9 where patterns should look familiar from our description of the C[2]-mim^+^-based systems. For each of the ILs we report on here, Figure 9 (left) shows two peaks and one antipeak. The red peak corresponding to the polar correlations occurs at the lowest *q* value highlighting that the charged slab is further away from the interface than the apolar layer associated with the black line; the green line associated with polar–apolar correlations shows as an antipeak because there is an offset between the two types of moieties.

Figure 9 (right) shows a more detailed picture of the contribution of correlations associated with different pairs of species. For example, for a given IL we see that cation head (positive–positive) and anion head (negative–negative) correlations always contribute at about the same *q* value to  and so does the positive–negative correlation. This implies that the depth along *z* of cation heads and anion heads is in sync on a charge neutral slab (or multiple slabs if there is more than one polar peak in the real space number density); tail–tail correlations are shifted to larger *q* values with respect to these (i.e., tails are closer to the vacuum interface). Notice that alkyl–alkyl, alkyl–fluoroalkyl, and fluoroalkyl–fluoroalkyl correlations also show as peaks but not necessarily at the exact same *q* values. This is because fluoroalkyl tails are in most cases of different length than alkyl tails and both are necessarily anchored to the charge slab. In all cases and as expected, the charge–alkyl and charge–fluoroalkyl correlations show as antipeaks because of the charge and tail interface-induced spatial offset. We can now appreciate how, in contrast to the case of the C[2]-mim^+^-based ILs, there is no trend for the C[8]-mim^+^-based ILs in Figure 6 (right) because the first apolar slab width is less affected by changing the anion. Instead, the behavior is very complex involving the interplay of multiple different subspecies correlations and X-ray form factors giving them specific weights.

## 4 Conclusions and Final Remarks

The organization of ionic liquids with alkyl and fluoroalkyl moieties at the vacuum interface is defined by what one could call chargephobicity, or the tendency of charge to be separated from the interface by the thickest possible single ion apolar tail layer. For the systems studied here, this can be either the alkyl tail of C[8]-mim^+^ or the fluoroalkyl tail of anions. Positive and negative charge species organize for the best geometry in slabs that lead to overall charge neutrality. Because of this, the atomic species at the immediate interface may be alkyl or fluoroalkyl, depending on which tail (cationic or anionic) is longest, because these are necessarily anchored to the charged slab. Because lateral charge alternation is the main organizational pattern, alkyl and fluoroalkyl tails must adjacently coexist on a molecular level. The persistence length scale of oscillations at and away from the interface depends on the system, but all are less than ∼7 nm. Not all tails associated with the charge slab point outward toward the vacuum interface, and in fact those pointing inward can give rise to an incipient internal double layer depending on the system. If one was to experimentally study these systems using the X-ray reflectivity technique, it is our prediction that the Fresnel normalized reflectivity will appear as a featureless inverse sigmoid looking function. Whereas a clear trend exists for the normalized reflectivity as a function of fluoroalkyl tail length for the C[2]-mim^+^-based ILs, we see no clear trend for those based on C[8]-mim^+^ for which the first apolar layer thickness does not vary much with a change in anion and multiple other factors enter into play. The peaks and antipeaks analysis of reflectivity splits this intensity into the contribution of intensities of pair subcomponents. Similar to the partial subcomponents in the bulk *S*(*q*) for ionic liquids,  shows the two peaks and one antipeak signature of polar–apolar oscillations. These real-space oscillations are not the randomly oriented bulk density fluctuations resulting from the naturally forming polar and apolar domains, but instead are due to the break in symmetry at the interface. What is significantly different in reflectivity with respect to the bulk *S*(*q*) is that there are no major peaks and antipeaks associated with positive–negative charge alternation. This is simply because this type of ordering is not along the *z*-direction (to which specular reflectivity is sensitive to) but instead in lateral slabs along the *x*,*y*-planes. When considering the polarity partition of reflectivity we always see that the polar–polar peak appears at lower *q* value than the apolar–apolar peak. This simply means that the polar slab is deeper inside the bulk than the apolar layer. Splitting our analysis into the contribution of positive, negative, alkyl, and fluoroalkyl pair of moieties, we further learn that anion related correlations can become dominant because of the significant weight provided by X-ray form factors.

## References

[ref1] Gannon T. J.; Law G.; Watson P. R.; Carmichael A. J.; Seddon K. R. First Observation of Molecular Composition and Orientation at the Surface of a Room-Temperature Ionic Liquid. Langmuir 1999, 15, 8429–8434. 10.1021/la990589j.

[ref2] Sloutskin E.; Ocko B. M.; Tamam L.; Kuzmenko I.; Gog T.; Deutsch M. Surface Layering in Ionic Liquids: An X-ray Reflectivity Study. J. Am. Chem. Soc. 2005, 127, 7796–7804. 10.1021/ja0509679. 15913369

[ref3] Bowers J.; Vergara-Gutierrez M. C.; Webster J. R. P. Surface Ordering of Amphiphilic Ionic Liquids. Langmuir 2004, 20, 309–312. 10.1021/la035495v. 15743071

[ref4] Baldelli S. Influence of Water on the Orientation of Cations at the Surface of a Room-Temperature Ionic Liquid:A Sum Frequency Generation Vibrational Spectroscopic Study. J. Phys. Chem. B 2003, 107, 6148–6152. 10.1021/jp027753n.

[ref5] Rivera-Rubero S.; Baldelli S. Influence of Water on the Surface of Hydrophilic and Hydrophobic Room-Temperature Ionic Liquids. J. Am. Chem. Soc. 2004, 126, 11788–11789. 10.1021/ja0464894. 15382902

[ref6] Camci M. T.; Ulgut B.; Kocabas C.; Suzer S. XPS investigation of the vacuum interface of an ionic liquid under triangular electrical excitation for slow transients. Anal. Meth. 2018, 10, 4225–4228. 10.1039/C8AY01488D.

[ref7] Hayes R.; Warr G. G.; Atkin R. Structure and Nanostructure in Ionic Liquids. Chem. Rev. 2015, 115, 6357–6426. 10.1021/cr500411q. 26028184

[ref8] Als-Nielsen J.; McMorrow D. Elements of Modern X-ray Physics; John Wiley & Sons, Inc.: New York, 2011.

[ref9] Wu F.; Karunaratne W. V.; Margulis C. J. Ionic Liquid Mixture at the Vacuum Interface and the Peaks and Antipeaks Analysis of X-ray Reflectivity. J. Phys. Chem. C 2019, 123, 4914–4925. 10.1021/acs.jpcc.8b11958.

[ref10] Karunaratne W. V.; Sharma S.; Ocko B. M.; Margulis C. J. Structure of Molten Alkali Chlorides at Charged Interfaces and the Prediction and Interpretation of Their X-ray Reflectivity. J. Phys. Chem. C 2021, 125, 25227–25242. 10.1021/acs.jpcc.1c07135.

[ref11] Jurado L. A.; Kim H.; Arcifa A.; Rossi A.; Leal C.; Spencer N. D.; Espinosa-Marzal R. M. Irreversible structural change of a dry ionic liquid under nanoconfinement. Phys. Chem. Chem. Phys. 2015, 17, 13613–13624. 10.1039/C4CP05592F. 25941682

[ref12] Parr D.; Chrestenson J.; Malik M. K.; Molter M.; Zibart C.; Egan B.; Haverhals L. M. Structure and Dynamics at Ionic Liquid/Electrode Interfaces. ECS Trans. 2015, 66, 35–42. 10.1149/06630.0035ecst.

[ref13] Anaredy R. S.; Shaw S. K. Long-Range Ordering of Ionic Liquid Fluid Films. Langmuir 2016, 32, 5147–5154. 10.1021/acs.langmuir.6b00304. 27138261

[ref14] Shin J. Y.; Yamada S. A.; Fayer M. D. Dynamics of a Room Temperature Ionic Liquid in Supported Ionic Liquid Membranes vs the Bulk Liquid: 2D IR and Polarized IR Pump–Probe Experiments. J. Am. Chem. Soc. 2017, 139, 311–323. 10.1021/jacs.6b10695. 27973786

[ref15] Shin J. Y.; Yamada S. A.; Fayer M. D. Carbon Dioxide in a Supported Ionic Liquid Membrane: Structural and Rotational Dynamics Measured with 2D IR and Pump–Probe Experiments. J. Am. Chem. Soc. 2017, 139, 11222–11232. 10.1021/jacs.7b05759. 28723129

[ref16] Nishida J.; Breen J. P.; Wu B.; Fayer M. D. Extraordinary Slowing of Structural Dynamics in Thin Films of a Room Temperature Ionic Liquid. ACS Cent. Sci. 2018, 4, 1065–1073. 10.1021/acscentsci.8b00353. 30159404 PMC6107873

[ref17] Haddad J.; Pontoni D.; Murphy B. M.; Festersen S.; Runge B.; Magnussen O. M.; Steinrück H.-G.; Reichert H.; Ocko B. M.; Deutsch M. Surface Structure Evolution in a Homologous Series of Ionic Liquids. Proc. Natl. Acad. Sci. U. S. A 2018, 115, 1100–1107. 10.1073/pnas.1716418115. PMC5819424 29358372

[ref18] Mars J.; Hou B.; Weiss H.; Li H.; Konovalov O.; Festersen S.; Murphy B. M.; Rütt U.; Bier M.; Mezger M. Surface Induced Smectic Order in Ionic Liquids-an X-ray Reflectivity Study of [C_22_C_1_im]^+^[NTf_2_]^−^. Phys. Chem. Chem. Phys. 2017, 19, 26651–26661. 10.1039/C7CP04852A. 28960006

[ref19] Brkljača Z.; Klimczak M.; Miličević Z.; Weisser M.; Taccardi N.; Wasserscheid P.; Smith D. M.; Magerl A.; Smith A.-S. Complementary Molecular Dynamics and X-ray Reflectivity Study of an Imidazolium-Based Ionic Liquid at a Neutral Sapphire Interface. J. Phys. Chem. Lett. 2015, 6, 549–555. 10.1021/jz5024493. 26261977

[ref20] Karunaratne W. V.; Margulis C. J. Structure and Dynamics of an Ionic Liquid Mixture Film Confined by Mica. J. Phys. Chem. C 2019, 123, 20971–20979. 10.1021/acs.jpcc.9b05510.

[ref21] Hettige J. J.; Amith W. D.; Castner E. W.; Margulis C. J. Ionic Liquids with Symmetric Diether Tails: Bulk and Vacuum-Liquid Interfacial Structures. J. Phys. Chem. B 2017, 121, 174–179. 10.1021/acs.jpcb.6b09148. 28005353

[ref22] Amith W. D.; Hettige J. J.; Castner E. W. Jr.; Margulis C. J. Structures of Ionic Liquids Having Both Anionic and Cationic Octyl Tails: Lamellar Vacuum Interface vs Sponge-Like Bulk Order. J. Phys. Chem. Lett. 2016, 7, 3785–3790. 10.1021/acs.jpclett.6b01763. 27607189

[ref23] Aliaga C.; Baldelli S. A Sum Frequency Generation Study of the Room-Temperature Ionic Liquid-Titanium Dioxide Interface. J. Phys. Chem. C 2008, 112, 3064–3072. 10.1021/jp709753r.

[ref24] Baldelli S.; Bao J.; Wu W.; Pei S.-s. Sum frequency generation study on the orientation of room-temperature ionic liquid at the graphene–ionic liquid interface. Chem. Phys. Lett. 2011, 516, 171–173. 10.1016/j.cplett.2011.09.084.

[ref25] Xu S.; Xing S.; Pei S.-S.; Baldelli S. Sum Frequency Generation Spectroscopy Study of an Ionic Liquid at a Graphene-BaF2 (111) Interface. J. Phys. Chem. B 2014, 118, 5203–5210. 10.1021/jp502500u. 24785472

[ref26] Xu S.; Xing S.; Pei S.-S.; Ivaništšev V.; Lynden-Bell R.; Baldelli S. Molecular Response of 1-Butyl-3-Methylimidazolium Dicyanamide Ionic Liquid at the Graphene Electrode Interface Investigated by Sum Frequency Generation Spectroscopy and Molecular Dynamics Simulations. J. Phys. Chem. C 2015, 119, 26009–26019. 10.1021/acs.jpcc.5b08736.

[ref27] Peñalber C. Y.; Baldelli S. Observation of Charge Inversion of an Ionic Liquid at the Solid Salt–Liquid Interface by Sum Frequency Generation Spectroscopy. J. Phys. Chem. Lett. 2012, 3, 844–847. 10.1021/jz3000917. 26286408

[ref28] Perkin S.; Salanne M.; Madden P.; Lynden-Bell R. Is a Stern and diffuse layer model appropriate to ionic liquids at surfaces?. Proc. Natl. Acad. Sci. U. S. A. 2013, 110, E4121 10.1073/pnas.1314188110. 24135005 PMC3816423

[ref29] Gebbie M. A.; Smith A. M.; Dobbs H. A.; Lee A. A.; Warr G. G.; Banquy X.; Valtiner M.; Rutland M. W.; Israelachvili J. N.; Perkin S.; Atkin R. Long range electrostatic forces in ionic liquids. Chem. Commun. 2017, 53, 1214–1224. 10.1039/C6CC08820A. 28000809

[ref30] Elbourne A.; Sweeney J.; Webber G. B.; Wanless E. J.; Warr G. G.; Rutland M. W.; Atkin R. Adsorbed and near-surface structure of ionic liquids determines nanoscale friction. ChemComm 2013, 49, 6797. 10.1039/c3cc42844c 23784030

[ref31] Elbourne A.; Voitchovsky K.; Warr G. G.; Atkin R. Ion structure controls ionic liquid near-surface and interfacial nanostructure. Chem. Sci. 2015, 6, 527–536. 10.1039/C4SC02727B. 28936307 PMC5588538

[ref32] Zhang Y.; Rutland M. W.; Luo J.; Atkin R.; Li H. Potential-Dependent Superlubricity of Ionic Liquids on a Graphite Surface. J. Phys. Chem. C 2021, 125, 3940–3947. 10.1021/acs.jpcc.0c10804.

[ref33] Li H.; Zhang Y.; Jones S.; Segalman R.; Warr G. G.; Atkin R. Interfacial nanostructure and friction of a polymeric ionic liquid-ionic liquid mixture as a function of potential at Au(1 1 1) electrode interface. J. Colloid Interface Sci. 2022, 606, 1170–1178. 10.1016/j.jcis.2021.08.067. 34487936

[ref34] Zhao M.; Wu B.; Lall-Ramnarine S. I.; Ramdihal J. D.; Papacostas K. A.; Fernandez E. D.; Sumner R. A.; Margulis C. J.; Wishart J. F.; Castner E. W. Jr. Structural analysis of ionic liquids with symmetric and asymmetric fluorinated anions. J. Chem. Phys. 2019, 151, 074504 10.1063/1.5111643. 31438705

[ref35] Hess B.; Kutzner C.; van der Spoel D.; Lindahl E. GROMACS 4: Algorithms for Highly Efficient, Load-Balanced, and Scalable Molecular Simulation. J. Chem. Theory Comput. 2008, 4, 435–447. 10.1021/ct700301q. 26620784

[ref36] van der Spoel D.; Lindahl E.; Hess B.; Groenhof G.; Mark A. E.; Berendsen H. J. C. GROMACS: Fast, flexible, and free. J. Comput. Chem. 2005, 26, 1701–1718. 10.1002/jcc.20291. 16211538

[ref37] Abraham M. J.; Murtola T.; Schulz R.; Páll S.; Smith J. C.; Hess B.; Lindahl E. GROMACS: High performance molecular simulations through multi-level parallelism from laptops to supercomputers. SoftwareX 2015, 1–2, 19–25. 10.1016/j.softx.2015.06.001.

[ref38] Canongia Lopes J. N.; Deschamps J.; Pádua A. A. H. Modeling Ionic Liquids Using a Systematic All-Atom Force Field. J. Phys. Chem. B 2004, 108, 2038–2047. 10.1021/jp0362133.

[ref39] Canongia Lopes J. N.; Pádua A. A. H. Molecular Force Field for Ionic Liquids Composed of Triflate or Bistriflylimide Anions. J. Phys. Chem. B 2004, 108, 16893–16898. 10.1021/jp0476545.

[ref40] Canongia Lopes J. N.; Padua A. A. H.; Shimizu K. Molecular Force Field for Ionic Liquids IV: Trialkylimidazolium and Alkoxycarbonyl-Imidazolium Cations; Alkylsulfonate and Alkylsulfate Anions. J. Phys. Chem. B 2008, 112, 5039–5046. 10.1021/jp800281e. 18380506

[ref41] Shimizu K.; Almantariotis D.; Gomes M. F. C.; Pádua A. A. H.; Lopes J. N. C. Molecular Force Field for Ionic Liquids V: Hydroxyethylimidazolium, Dimethoxy-2- Methylimidazolium, and Fluoroalkylimidazolium Cations and Bis(Fluorosulfonyl)Amide, Perfluoroalkanesulfonylamide, and Fluoroalkylfluorophosphate Anions. J. Phys. Chem. B 2010, 114, 3592–3600. 10.1021/jp9120468. 20175555

[ref42] Jorgensen W. L.; Maxwell D. S.; Tirado-Rives J. Development and Testing of the OPLS All-Atom Force Field on Conformational Energetics and Properties of Organic Liquids. J. Am. Chem. Soc. 1996, 118, 11225–11236. 10.1021/ja9621760.

[ref43] McDonald N. A.; Jorgensen W. L. Development of an All-Atom Force Field for Heterocycles. Properties of Liquid Pyrrole, Furan, Diazoles, and Oxazoles. J. Phys. Chem. B 1998, 102, 8049–8059. 10.1021/jp981200o.

[ref44] Price M. L. P.; Ostrovsky D.; Jorgensen W. L. Gas-Phase and Liquid-State Properties of Esters, Nitriles, and Nitro Compounds with the OPLS-AA Force Field. J. Comput. Chem. 2001, 22, 1340–1352. 10.1002/jcc.1092.

[ref45] Watkins E. K.; Jorgensen W. L. Perfluoroalkanes: Conformational Analysis and Liquid-State Properties from ab Initio and Monte Carlo Calculations. J. Phys. Chem. A 2001, 105, 4118–4125. 10.1021/jp004071w.

[ref46] Hockney R.; Goel S.; Eastwood J. Quiet High-Resolution Computer Models of a Plasma. J. Comput. Phys. 1974, 14, 148–158. 10.1016/0021-9991(74)90010-2.

[ref47] Darden T.; York D.; Pedersen L. Particle mesh Ewald: An N*log(N) method for Ewald sums in large systems. J. Chem. Phys. 1993, 98, 10089–10092. 10.1063/1.464397.

[ref48] Essmann U.; Perera L.; Berkowitz M. L.; Darden T.; Lee H.; Pedersen L. G. A smooth particle mesh Ewald method. J. Chem. Phys. 1995, 103, 8577–8593. 10.1063/1.470117.

[ref49] Song W.; Rossky P. J.; Maroncelli M. Modeling alkaneperfluoroalkane interactions using all-atom potentials: Failure of the usual combining rules. J. Chem. Phys. 2003, 119, 9145–9162. 10.1063/1.1610435.

[ref50] Agilio Padua, FFTtool v1.0.0. 2015; https://zenodo.org/record/18618.

[ref51] Martinez L.; Andrade R.; Birgin E. G.; Martinez J. M. PACKMOL: A package for building initial configurations for molecular dynamics simulations. J. Comput. Chem. 2009, 30, 2157–2164. 10.1002/jcc.21224. 19229944

[ref52] Bussi G.; Donadio D.; Parrinello M. Canonical Sampling Through Velocity Rescaling. J. Chem. Phys. 2007, 126, 014101 10.1063/1.2408420. 17212484

[ref53] Berendsen H. J. C.; Postma J. P. M.; van Gunsteren W. F.; DiNola A.; Haak J. R. Molecular dynamics with coupling to an external bath. J. Chem. Phys. 1984, 81, 3684–3690. 10.1063/1.448118.

[ref54] Nosé S. A unified formulation of the constant temperature molecular dynamics methods. J. Chem. Phys. 1984, 81, 511–519. 10.1063/1.447334.

[ref55] Nosé S. A molecular dynamics method for simulations in the canonical ensemble. Mol. Phys. 1984, 52, 255–268. 10.1080/00268978400101201.

[ref56] Hoover W. G. Canonical dynamics: Equilibrium phase-space distributions. Phys. Rev. A 1985, 31, 1695–1697. 10.1103/PhysRevA.31.1695. 9895674

[ref57] Parrinello M.; Rahman A. Polymorphic transitions in single crystals: A new molecular dynamics method. J. Appl. Phys. 1981, 52, 7182–7190. 10.1063/1.328693.

[ref58] Yeh I.-C.; Berkowitz M. L. Ewald summation for systems with slab geometry. J. Chem. Phys. 1999, 111, 3155–3162. 10.1063/1.479595.

[ref59] Yeh I.-C.; Wallqvist A. On the proper calculation of electrostatic interactions in solid-supported bilayer systems. J. Chem. Phys. 2011, 134, 055109 10.1063/1.3548836. 21303169

