# Supplementary material for: Vacuum Interfacial Structure and X-ray Reflectivity of Imidazolium-Based Ionic Liquids with Perfluorinated Anions from a Theory and Simulations Perspective
Source: J Phys Chem C Nanomater Interfaces. 2022 Aug 5;126(32):13936–45. doi: 10.1021/acs.jpcc.2c03311 (PMC9394757; doi:10.1021/acs.jpcc.2c03311)
Supplement: Supplementary file 1 — jp2c03311_si_001.pdf [file jp2c03311_si_001.pdf]

Supporting Information: Vacuum Interfacial Structure and X-ray Reflectivity of Imidazolium-Based Ionic Liquids with Perfluorinated Anions from a Theory and Simulations Perspective

Waruni V. Karunaratne,[†] Man Zhao,[‡] Edward W. Castner, Jr.,[‡] and Claudio J. Margulis[*,†]

†*Department of Chemistry, University of Iowa, Iowa City, IA 52242, USA*

‡*Department of Chemistry and Chemical Biology, Rutgers, The State University of New Jersey, Piscataway, New Jersey 08854, USA*

E-mail: claudio-margulis@uiowa.edu

S.1 Side View of Selected Systems Matching Figure 2

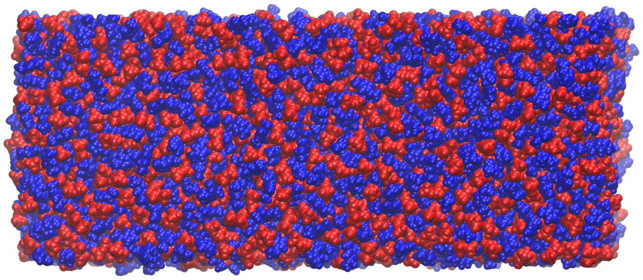

(a)

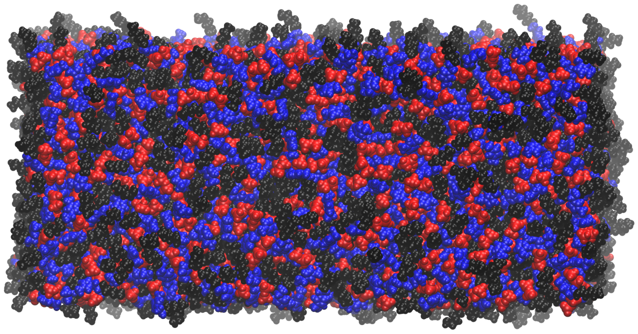

(b)

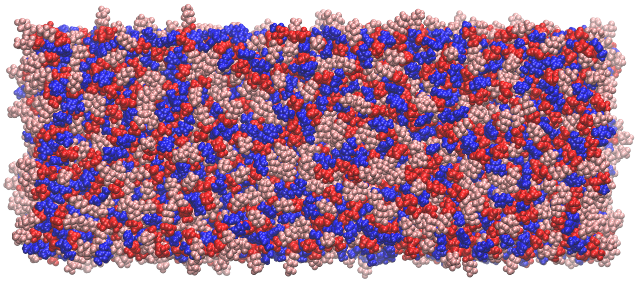

(c)

Figure S.1: Side view of final simulation snapshots (i.e. z-axis of the simulation box lies parallel to the page) for vacuum confined (a) C[2]-mim$^+$/FSI$^-$, (b) C[8]-mim$^+$/FSI$^-$, and (c) C[2]-mim$^+$/BSI(1,6)$^-$. For all plots the color convention is positive: blue, negative: red, alkyl: black and fluoroalkyl: rose.

S.2 Comparison of Structure and Dynamics between Experiments and Simulations

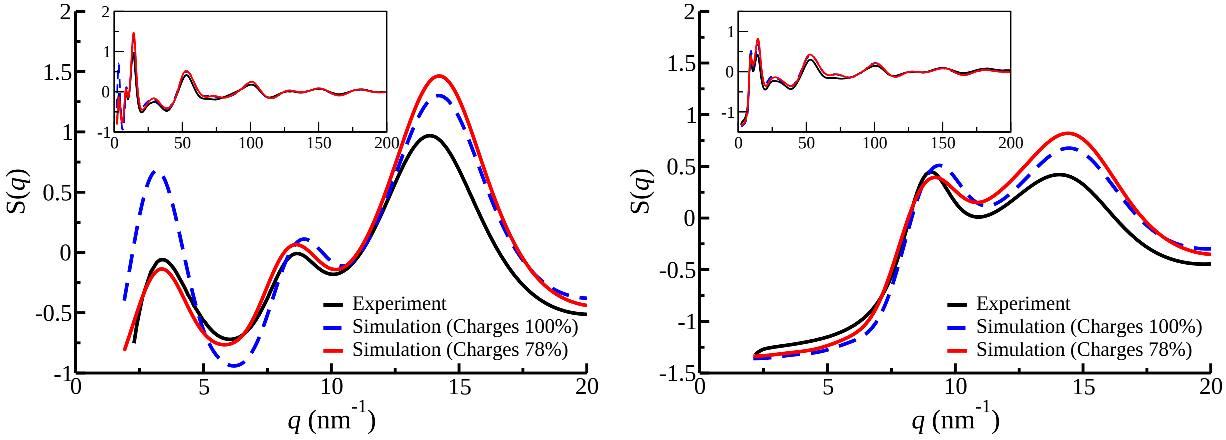

(a) (b)

Figure S.2: A comparison of computed vs. experimental structure functions $S(q)$ at 298 K for (a)C[8]-mim$^+$/NTf$_2^-$ and (b)C[2]-mim$^+$/NTf$_2^-$. For these calculations, cubic simulation boxes containing 500 ion pairs were prepared, energy minimized, and equilibrated following the same protocol as that described in Section 2 under *Bulk ILs Simulations* except that the final target temperature was 298 K. The last 20 ns of the 100 ns production run was used to compute $S(q)$; experimental $S(q)$ are from reference 1.

Table S.1: Densities at 298 K

IL System	Density (g cm^{-3})		
	Experiment[2]	Simulation (Charges 100%)	Simulation (Charges 78%)
C[8]-mim$^+$/NTf$_2^-$	1.322	1.37904	1.35623
C[2]-mim$^+$/NTf$_2^-$	1.522	1.59815	1.55758

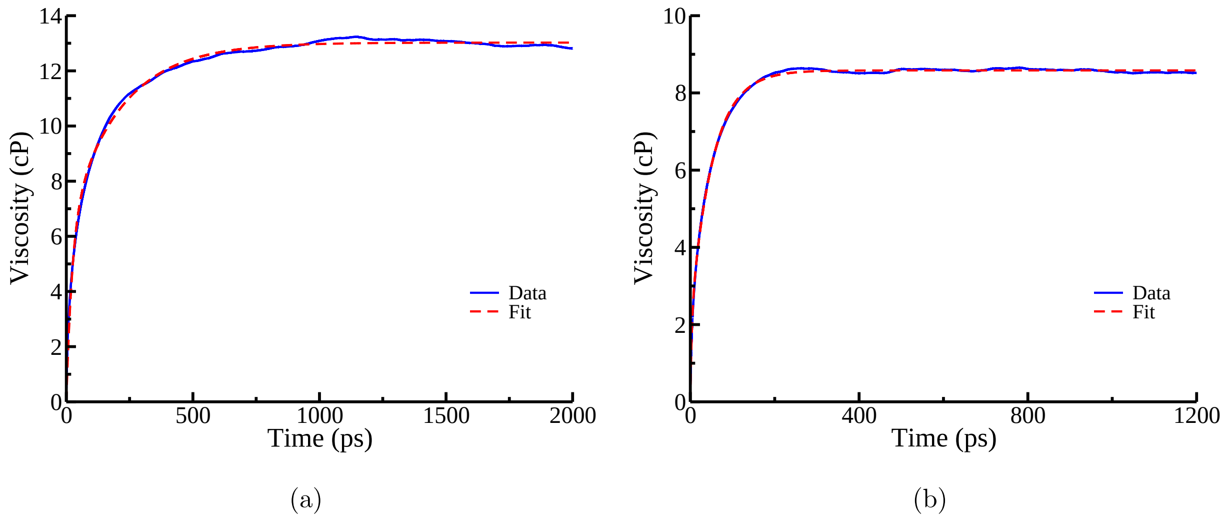

Figure S.3: Viscosities computed using the stress tensor autocorrelation function formalism for (a)C[8]-mim$^+$/NTf$_2^-$ and (b)C[2]-mim$^+$/NTf$_2^-$. For these calculations, cubic simulation boxes containing 500 ion pairs were prepared and equilibrated similarly to the way described in Section 2 under *Bulk ILs Simulations* with scaled charges at 78% and target temperature of 375 K, except that instead of an NPT step we run a 20 ns NVE step from which the last frame was used to launch multiple parallel NVT simulations with different random initial velocities. The temperature used for the NVT runs is the average of that obtained in the NVE runs and the viscosity was computed from this ensemble of simulations each 7 ns in duration using a modified version of the protocol in reference 3. Cumulative integrals of the Green-Kubo expression for the viscosity were fitted to a double exponential to get the average viscosity value at long time; see Table S.2.

Table S.2: Viscosities of C[8]-mim$^+$/NTf$_2^-$ and C[2]-mim$^+$/NTf$_2^-$ IL systems

IL System	Temperature (K)	Viscosity (cP)	
		Experiment[4]	Simulation-Charges 78% (#NVT trajectories)
C[8]-mim$^+$/NTf$_2^-$	372.259	8.52	13.02 (100)
C[2]-mim$^+$/NTf$_2^-$	373.218	5.77	8.58 (80)

S.3 Simulation System Sizes

Table S.3: Size of bulk and interfacial (bulk+vacuum) simulation boxes.

IL System	LX=LY (nm)	LZ_{bulk} (nm)	$LZ_{interface}$ (nm)
C[2]-mim$^+$/FSI$^-$	6.60911	16.25747	65.02988
C[2]-mim$^+$/NTf_2^-	7.19547	17.69983	70.79932
C[2]-mim$^+$/BETI$^-$	7.48230	19.69026	78.76104
C[2]-mim$^+$/BSI(1,4)$^-$	7.67427	20.19543	80.78172
C[2]-mim$^+$/BSI(1,6)$^-$	8.10346	21.00899	84.03596
C[2]-mim$^+$/BSI(1,8)$^-$	7.02328	31.60473	126.41892
C[8]-mim$^+$/FSI$^-$	7.81464	17.58293	70.33172
C[8]-mim$^+$/NTf_2^-	8.06903	19.69226	78.76904
C[8]-mim$^+$/BETI$^-$	7.32874	27.48274	109.83340
C[8]-mim$^+$/BSI(1,4)$^-$	7.02508	31.61288	126.61672
C[8]-mim$^+$/BSI(1,6)$^-$	8.44579	24.47361	97.89444
C[8]-mim$^+$/BSI(1,8)$^-$	6.75248	42.20303	168.81212

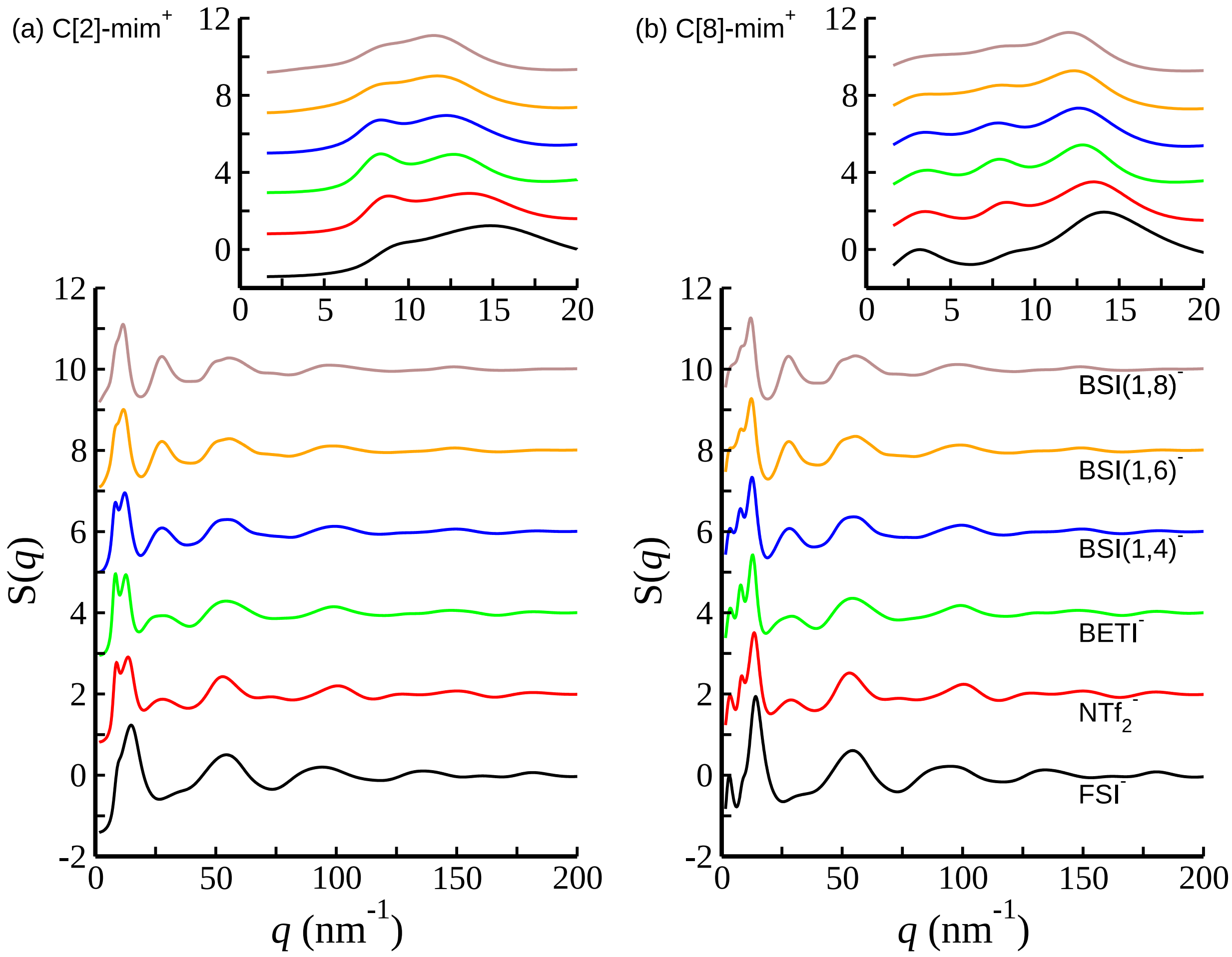

Figure S.4: Bulk structure functions $S(q)$ computed for (a) C[2]-mim$^+$-based ILs and (b) C[8]-mim$^+$-based ILs at 425 K with insets zooming into the low-q region. All plots are offset vertically for clarity.

S.4 Interfacial Number Density and X-ray reflectivity Profiles

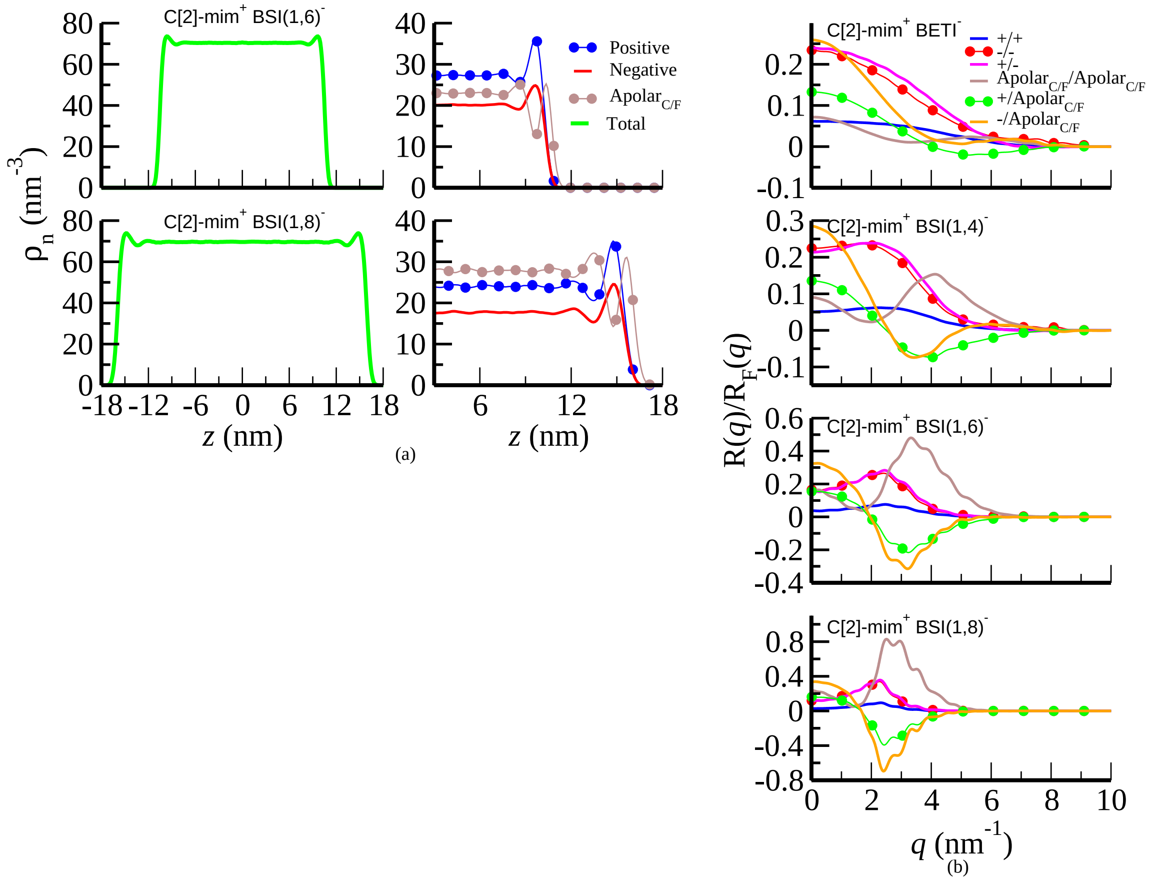

Figure S.5: (a left) Number density profiles for C[2]-mim$^+$/BSI(1,6)$^-$ and C[2]-mim$^+$/BSI(1,8)$^-$; (a right) for the same ILs, positive, negative and fluorocarbon number densities. (b) For C[2]-mim$^+$-based ILs with a fluoroalkyl tail as defined in Figure 1, partial subcomponents of the Fresnel-normalized X-ray reflectivity $\frac{R(q)}{R_F(q)}$ using the charge partition described in Equation 2.

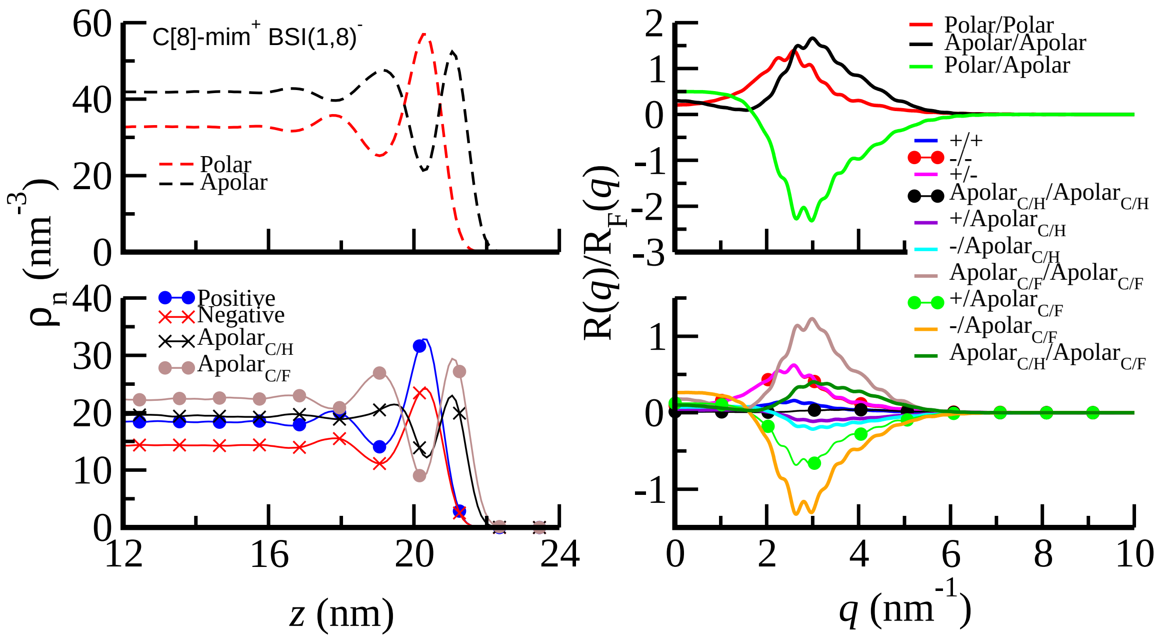

Figure S.6: (Left) Symmetrized number density profiles for C[8]-mim$^+$/BSI(1,8)$^-$ with '0' in the x-axis at the center of the simulation box; top and bottom show different splittings of the density based on the definitions in Figure 1. (Right) top and bottom show partial subcomponents of the Fresnel-normalized X-ray reflectivity $\frac{R(q)}{R_F(q)}$ for C[8]-mim$^+$/BSI(1,8)$^-$ based on Equations 1 and 2 respectively.

References

(1) Zhao, M.; Wu, B.; Lall-Ramnarine, S. I.; Ramdihal, J. D.; Papacostas, K. A.; Fernandez, E. D.; Sumner, R. A.; Margulis, C. J.; Wishart, J. F.; Castner, E. W., Jr. Structural analysis of ionic liquids with symmetric and asymmetric fluorinated anions. *J. Chem. Phys.* **2019**, *151*, 074504.

(2) Tokuda, H.; Hayamizu, K.; Ishii, K.; Susan, M. A. B. H.; Watanabe, M. Physicochemical Properties and Structures of Room Temperature Ionic Liquids. 2. Variation of Alkyl Chain Length in Imidazolium Cation. *J. Phys. Chem. B* **2005**, *109*, 6103–6110.

(3) Humbert, M. T.; Zhang, Y.; Maginn, E. J. PyLAT: Python LAMMPS Analysis Tools. *J. Chem. Inf. Model.* **2019**, *59*, 1301–1305.

(4) Tariq, M.; Carvalho, P. J.; Coutinho, J. A. P.; Marrucho, I. M.; Canongia Lopes, J. N.; Rebelo, L. P. N. Viscosity of (C_2-C_{14}) 1-alkyl-3-methylimidazolium bis(trifluoromethylsulfonyl)amide ionic liquids in an extended temperature range. *Fluid Phase Equilibr.* **2011**, *301*, 22–32.
